# A head-to-head comparison of S100B and GFAP/UCH-L1 for detection of traumatic intracranial lesions in a Scandinavian trauma cohort

**DOI:** 10.1186/s13049-025-01364-9

**Published:** 2025-03-25

**Authors:** Thea Overgaard Wichmann, Ayad Babaee, Kirsten Duch, Mikkel Mylius Rasmussen, Maj Lesbo, Ole Brink, Lars C. Borris, Claus V. B. Hviid

**Affiliations:** 1 Department of Neurosurgery, Aarhus University Hospital, Aarhus, Denmark; 2 Department of Surgery and Intensive Care, Viborg Regional Hospital, Viborg, Denmark; 3 Department of Clinical Biochemistry, Aalborg University Hospital, Aalborg, Denmark; 4 Research Data and Biostatistics, Aalborg University Hospital, Aalborg, Denmark; 5 Department of Clinical Medicine, Aarhus University, Aarhus, Denmark; 6 Department of Orthopaedic Surgery, Viborg Regional Hospital, Viborg, Denmark; 7 Department of Orthopaedic Surgery, Aarhus University Hospital, Aarhus, Denmark; 8 Department of Clinical Medicine, Aalborg University, Aalborg, Denmark

**Keywords:** Mild traumatic brain injury (mTBI), Glial fibrillary acidic protein (GFAP), S100 astroglial calcium-binding protein B (S100B), Ubiquitin C-terminal hydrolase-L1 (UCH-L1)

## Abstract

**Background:**

Few countries recommend glial fibrillary protein (GFAP) and ubiquitin C-terminal hydrolase-L1 (UCH-L1) as a substitute for S100 astroglial calcium-binding protein B (S100B) in early detection of traumatic intracranial lesions in mild TBI (mTBI). This study aims to evaluate the classification agreement between S100B and GFAP/UCH-L1 in a Scandinavian trauma cohort, to evaluate the performance characteristics of S100B and GFAP/UCH-L1 for detection of traumatic intracranial lesions, and lastly to evaluate the laboratory performance of the GFAP/UCH-L1 assay.

**Methods:**

Prospectively collected data from an unselected cohort of 379 adult trauma patients admitted to a level I trauma center at Aarhus University Hospital, Denmark, were retrospectively analyzed. Analyses were performed in the unselected cohort, in sub-cohort 1 (n = 218) i.e. patients with any evidence of TBI in their chart as well as in sub-cohort 2 (n = 105) i.e. patients with mTBI defined as Glasgow Coma Scale score ≥ 14, an injury severity score ≤ 15, and blood sampling within 6 h or 12 h after trauma. Plasma-samples were used for GFAP/UCH-L1 measurement and serum-samples were used for S100B measurement. Data analysis involved agreement analysis using Cohens kappa and sensitivity, specificity, positive predictive value and negative predictive value for each biomarker in each of the three cohorts. Lastly, levels of GFAP/UCH-L1 measured by the Alinity-I platform and the Simoa platform were compared.

**Results:**

Classification agreement between GFAP/UCH-L1 and S100B was high in all three cohorts, but Cohens kappa improved with increasing proximity to clinical biomarker use and reached an almost perfect identity in sub-cohort 2 (0.70, 95% CI 0.62–0.92). S100b had a sensitivity and negative predictive value of 100% in sub-cohort 2, while GFAP/UCH-L1 reached 100% across all three cohorts. The specificities for both S100B and GFAP/UCH-L1 were relatively low. Comparing GFAP/UCH-L1 levels between platforms revealed a low concordance with the Alinity-I platform measuring GFAP levels on average 65% lower and UCH-L1 levels 84% higher than the Simoa platform.

**Conclusions:**

In this study, S100B and GFAP/UCH-L1 had an almost perfect agreement for classification of mTBI patients and comparable diagnostic performances for detecting traumatic intracranial lesions. Our results therefore support GFAP/UCH-L1 as a feasible alternative to S100B for detecting traumatic intracranial lesions in mTBI.

**Supplementary Information:**

The online version contains supplementary material available at 10.1186/s13049-025-01364-9.

## Background

Extensive research has been conducted to identify biomarkers for detection of traumatic intracranial lesions after mild traumatic brain injury (mTBI). TBI is a heterogenous disorder that range from mild concussions to severe injuries with life-threatening intracranial lesions. The majority of TBIs are mTBI that presents with a variety of symptoms [1]. Roughly 7% of patients with mTBI develop a traumatic intracranial lesion, but less than 1% of these lesions requires prompt neurosurgical intervention or lead to death [2]. While head computed tomography (CT) is the standard for rapid detection of intracranial lesions following moderate or severe TBI [3], the use of head-CT in mTBI is more complex. Despite clinical decision rules for head-CT following mTBI [4], non-adherence to guidelines is common leading to head-CT without guideline-based indication [5, 6]. Use of biomarkers as an additive to clinical decision rules might increase guideline-adherence, and thereby reduce unnecessary radiation exposure, emergency department time, and health care costs.

The Scandinavian Neurotrauma Committee (SNC) guidelines for management of minimal, mild and moderate head trauma in adults recommend the use of S100 astroglial calcium-binding protein B (S100B) as a screening tool for early detection of traumatic intracranial lesions in mTBI [7]. Single test of S100B can help determine the need for head-CT within 6 h of injury. The sensitivity and negative predictive value of a S100B single test in detecting traumatic intracranial lesions in adults is 97–100% and 92–100%, respectively [8–10]. Yet, S100B cannot be used in pediatric head trauma, with concomitant extracranial injuries or beyond 6 h after the injury, which challenge clinical implementation of S100B [7, 11]. The introduction of a combination test of glial fibrillary acidic protein (GFAP) and ubiquitin C-terminal hydrolase (UCH-L1) in mTBI have therefore gained much interest [12–16]. Both GFAP and UCH-L1 are detectible within 1 h of injury with peak levels at 20 h and 8 h, respectively [13–16]. The sensitivity and negative predictive value of the GFAP and UCH-L1 combination test in detecting traumatic intracranial lesions in adults is reported to be 97–100% and 99.6–100%, respectively [17, 18]. After the US Food and Drug Administration (FDA) approved GFAP and UCH-L1 for clinical use in adults with mTBI to help determine the need for CT scan within 12 h of injury [19], the French and Spanish guidelines for management of mTBI have incorporated the test [20, 21]. Only a preliminary study has evaluated the SNC guideline substituting S100B with GFAP [22]. Given limited Scandinavian data comparing S100B and GFAP/UCH-L1 as early screening tools in TBI, the primary endpoint of this study was to evaluate the level of agreement between S100B and GFAP/UCH-L1 in a Scandinavian trauma cohort. The secondary endpoint was to evaluate the performance characteristics of S100B and GFAP/UCH-L1 for detection of traumatic intracranial lesions. The third endpoint was to evaluate the performance of the GFAP/UCH-L1 assay.

## Methods

### Study cohort

This study was conducted on biobank material from the SURVIVE cohort [23, 24]. The SURVIVE cohort is an unselected cohort of 418 patients admitted to the level-I trauma center at Aarhus University Hospital, Denmark, between March 2017 and February 2018. Patients aged ≥ 18 years fulfilling the Advanced Trauma Life Support criteria for trauma team activation were considered eligible for inclusion. Patients were excluded from the study if pregnant, dead upon arrival, declining/withdrawing consent or if blood sampling proved impossible. Patients with multiple admission during the study period was only included once [23]. For the present study, 39 patients were excluded due to insufficient material for either S100B or GFAP/UCH-L1 analysis (n = 36) or because of inconclusive GFAP/UCH-L1 results and insufficient material for reanalysis (n = 3). Patients with TBI were identified by the description in the medical records recorded before any CT-scans and/or secondary survey. Descriptions of trauma to the head or face, wounds or injuries to the head and/or face, confirmed or suspected loss of consciousness following a relevant trauma, amnesia for the trauma, and suspicion of concussions were considered TBI. Information from medical records were extracted independently by two medical doctors and any disagreement (70 patients) was cross-checked by a third doctor with competences in trauma care.

### Clinical information

The SURVIVE cohort had clinical information collected retrospectively from the medical records and the local trauma registry. For the SURVIVE cohort, information on age, sex, time of hospital admission, advanced airway management on admission, and preexisting medical treatment was retrieved from the medical records, while information on abbreviated injury score, time of injury and mechanism of injury was retrieved from the trauma registry. Time from injury to admission and Injury Severity Score (ISS) [25] were calculated based on this information.

For the present study, information on TBI, Marshall score and Glasgow Coma Scale (GCS) on admission was retrieved from medical records. In five patients, the GCS was not described in the admission chart. Three of these patients were obviously unaffected from the trauma and were assigned a score of 15. One was described as completely unresponsive on scene and assigned a score of 3. In the last case, a GCS could not be safely judged from the charts and the patients was excluded from analysis involving GCS score. Patients intubated on admission were assigned last registered GCS score which was retrieved from the admission records or from the prehospital records. Data on GCS were extracted by one medical doctor and cross-checked by another medical doctor with special competences in neurosurgery. The Marshall CT Classification score was assigned based on admission head-CT. The Marshall classification score range from 1 to 6 i.e. a score of 1 is assigned to patients with no visible intracranial lesions, whereas patients with non-evacuated mass lesions are assigned a score of 6 [26]. Data on head-CT were evaluated by retrospective review of the trauma scan by a junior doctor with special competence in neurosurgery and verified by a senior consultant in neurosurgery.

### Blood sampling

The SURVIVE cohort had blood samples collected from an antecubital vein or an arterial cannula by a certified laboratory technician. Samples were collected upon arrival to the trauma center, and 15 ± 3 and 72 ± 6 h after arrival. Serum, lithium heparin- and EDTA-anticoagulated tubes (BD Vacutainer®, Becton Dickinson and Company, Franklin Lakes, NJ, USA) were used. Samples for routine biochemistry and hematology analysis were processed in our accredited laboratory according to standard procedures for clinical analysis. Samples for study analyses were process within 2 h of collection. Serum samples were allowed to clot for a minimum of 30 min at 22–24 °C before centrifugation. All samples were centrifuged at 3000 g for 25 min at 22–24 °C and frozen at 80 °C until further analysis.

### Laboratory analysis

#### Study laboratory analyses

The TBI® assay was established on our Alinity-i platform (Abbott, Abbott Park, Illinois, USA). The assay is an automated panel analysis with an analysis time of 18 min using proprietary reagents to measure GFAP and UCH-L1 by chemiluminescence technology. At the cut (GFAP < 35.0 ng/L and UCH-L1 < 400.0 ng/L), the assay has a sensitivity of 96.7% (95% CI 91.7–98.7) and a negative predictive value of 99.4% (95% conficence interval (CI) 98.6–99.8) for detection of traumatic intracranial lesions on head-CT in adults with mTBI within 12 h of injury [27]. The limit of detection is 3.2 ng/L and 18.3 ng/L for GFAP and UCH-L1, respectively. Measurement and linearity ranges are 6.1 to 42.000 ng/L for GFAP and 26.3 to 25.000 ng/L for UCH-L1. The intra-laboratory imprecisions for GFAP are 3.7% (level 25.9 ng/L), 3.3% (level 508.6 ng/L), and 3.9% (level 31,225.4 ng/L), and for UCH-L1 4.1% (level 247.9 ng/L), 3.0% (level 2047.3 ng/L), and 3.6% (level 15,310.9 ng/L). The reference intervals are 6.6 -70.9 ng/L for GFAP and 44.7 to 226.8 ng/L for UCH-L1. EDTA-anticoagulated samples were thawed at 22–24 °C and centrifugated at 2000 g for 5 min to remove debris. Samples were batched analyzed in random order over six days using one reagent LOT. The analysis was performed by two certified laboratory technicians at Aalborg University Hospital experienced in the Alinity-I analysis and blinded to any study information. Company controls as specified above were used. Twenty-two controls at each level were analyzed during the study period and the imprecisions for GFAP were 3.1% (level 24.1 ng/L), 2.5% (level 485.3 ng/L) and 2.1% (level 30,521.4 ng/L). The imprecisions for UCH-L1 were: 2.0% (level 258.9 ng/L), 1.8% (level 2042.1 ng/L), 1.9% (level 15,133.0 ng/L). Levels of GFAP/UCH-L1 measured by the TBI® assay was compared with levels measured by Single Molecule Array (Simoa). These data were obtained from serum samples measured by the Neurology 4-plex assay B kit using a Simoa HD1 analyzer (Quanterix Corp, MA, USA). Details on this analysis has been published previously [24].

S100B was analyzed on a Cobas 8000 e602 module validated for routine clinical use. The analysis has an analysis time of 18 min and is performed on serum samples that requires a minimum 30 min pre-incubation. At the cutoff (< 0.1 µg/L), the assay has a sensitivity of 98.8% (95% CI 96 to 100) and a negative predictive value of 99.7% (95% CI 99.1–100) for detection of traumatic intracranial lesions on head-CT in adults with mTBI within 6 h of the injury [28]. The limit of detection is 0.015 µg/L and the measurement range is 0.015 to 39 µg/L. In our laboratory, the analysis is under internal and external quality control. Its bias is − 1.9% at 0.19 µg/L and the intermediate precision is 3.3% at level 0.19 µg/L and 3.5% at level 2.43 µg/L. Serum samples were thawed at 22–24 °C and centrifugated at 2000 g for 5 min to remove debris. The samples were loaded in random order as a batch in our fully automated laboratory and analyzed among routine samples over 16 days. The samples were handled by laboratory technicians blinded to the study. Subsequently, the data were extracted from the laboratory information system.

#### Routine laboratory analyses

Hematologic parameters (Hemoglobin, hematocrit, leukocytes, and platelets) and coagulation biomarkers (INR, APTT, d-dimer, fibrinogen, AT) were analyzed at the Department of Clinical Biochemistry, Aarhus University Hospital using validate routine clinical assays and according to standard operating procedures in our accredited laboratory (DS/EN ISO 15189). All samples were analyzed within two hours of collection.

### Statistics

Data are presented as absolute numbers with percentages, means with standard deviation (SD), medians with interquartile ranges (IQR) or minimum and maximum values depending on the data distribution. All statistical analyses were performed in accordance with our pre-study analysis plan, and presented with 95% confidence intervals (CI) when relevant. The primary endpoint was agreement between GFAP/UCH-L1 and S100B. Agreement was evaluated by Cohens kappa [29] analysis in three cohorts with increasing proximity to clinical biomarker use. Kappa values range from − 1 indicating complete disagreement to 1 indicating complete agreement. The first cohort consisted of the unselected cohort of trauma patients. Sub-cohort 1 consisted patients with any evidence of TBI in their chart. Sub-cohort 2 consisted of mTBI patients defined by a GCS score ≥ 14, an ISS score ≤ 15, and blood sampling within 6 h (S100B) or 12 h (GFAP/UCH-L1) after trauma. The secondary endpoint was diagnostic performance of S100B and GFAP/UCH-L1 to detect traumatic intracranial lesions on head-CT. Sensitivity, specificity, positive predictive values and negative predictive values were calculated for each biomarker in each of the three cohorts. Sub-analyses were performed to evaluate biomarker disagreement and determine component (GFAP or UCHL1) that triggered a positive GFAP/UCH-L1 test. This evaluation was descriptively in a 3 × 3 table of positive/negative/missing values of GFAP and UCH-L1 of patients with a positive head-CT. The third endpoint was an explorative comparison of quantitative levels of GFAP and UCH-L1 measured by the Alinity-I platform and the Simoa platform. This was done by Passing Bablok regression analysis with Lin´s concordance correlation coefficient and by Bland–Altman plot analysis as recommended by Clinical & Laboratory Standards Institute (CLSI) [30]. All analysis were performed in STATA 18.0 and a p-value of. 0.05 was considered statistically significant.

## Results

### Study cohort

There were 379 patients enrolled in the unselected trauma cohort having blood samples available for biomarker analysis. Among patients in the unselected trauma cohort, 218 had evident TBI and 105 had mTBI defined by a GCS score ≥ 14, an ISS score ≤ 15. Detailed characteristics of the trauma cohort and the two sub-cohorts are outlined in Table 1. In the unselected trauma cohort, 80.4% of patients presented with a GCS score of 14 or 15 and more than 70% of the patients had minor or moderate trauma only (ISS ≤ 15). From the unselected trauma cohort, 8 patients with a GCS < 14 were not included in sub-cohort 2 due to spontaneous subarachnoid hemorrhage (n = 3), suicidal attempt by strangulation (n = 2), severe intoxications (n = 2), and transfer from a lower-level trauma center (n = 1). In all cohorts, more than 90% of the patients had a head-CT performed and positive head-CT were found in 35.8% and 16.2% of patients in sub-cohort 1 and 2, respectively. Median time from injury to admission was below one hour in all cohorts. Only few patients were admitted more than 6 or 12 h after their injury in the unselected trauma cohort and sub-cohort 1. Among patients in sub-cohort 2 with a traumatic intracranial lesion on head-CT, two (1.9%) patients required neurosurgical intervention i.e. a craniotomy due to an acute subdural hematoma and a decompressive craniectomy due to traumatic subarachnoid hemorrhage and edema. Two patients in sub-cohort 2 were intubated prehospitally on a GCS 14 and GCS 15 for neuroprotective purpose. Only one of these patients had a positive head-CT and had neurosurgical intervention performed.

**Table 1:** Characteristics of study cohort

	Unselected trauma cohort (n = 379)	Sub-cohort 1 (n = 218)	Sub-cohort 2 (n = 105)
Age, years, mean ± SD	46.2 ± 20.2	49.2 ± 21.1	46.1 ± 20.3
Sex			
Male, n (%)	255 (67.3%)	151 (69.3%)	67 (63.8%)
Female, n (%)	124 (32.7%)	67 (30.7%)	38 (36.2%)
Glasgow Coma scale, n (%)			
14–15	304 (80.4%)	152 (69.7%)	105 (100%)
9–13	35 (9.2%)	32 (14.7%)	0 (0%)
3–8	39 (10.3%)	34 (15.6%)	0 (0%)
Unknown	1 (0.1%)	0 (0%)	0 (0%)
Injury severity score, n (%)			
1–8	187 (49.3%)	98 (44.9%)	76 (72.4%)
9–15	82 (21.6%)	45 (20.6%)	29 (27.6%)
16–24	48 (12.7%)	35 (16.1%)	0 (0%)
≥ 25	51 (13.5%)	36 (16.5%)	0 (0%)
Unknown	11 (2.9%)	4 (1.8%)	0 (0%)
Mechanism of injury, n (%)			
Fall	104 (27.4%)	74 (33.9%)	29 (27.6%)
Traffic	124 (32.7%)	78 (35.8%)	40 (38.1%)
Violence (incl suicide attempt)	26 (6.9%)	10 (4.6%)	6 (5.7%)
Other (incl unknown)	125 (33.0%)	56 (25.7%)	30 (28.6%)
Antiplatelet medication, n (%)	25 (6.6%)	17 (7.8%)	5 (4.8%)
Anticoagulant medication, n (%)	22 (5.8%)	18 (8.3%)	7 (6.7%)
Advanced airway management, n (%)	47 (12.4%)	41 (18.8%)	2 (1.9%)
Time from injury to admission, minutes, median (IQR)	55.0 (38.2)	55.0 (39.0)	47.0 (34.0)
Admission delay > 6 h from injury, n (%)	7 (2.1%)	2 (1.0%)	0 (0%)
Admission delay > 12 h from injury, n (%)	5 (1.5%)	1 (1.0%)	0 (0%)
Head CT performed, n (%)	347 (91.6%)	216 (99.1%)	104 (99.1%)
Head-CT lesion, n (%)	81 (21.4%)	78 (35.8%)	17 (16.2%)
Marshall Score, n (%)			
1	266 (76.7%)	138 (63.3%)	87 (82.9%)
2–4	57 (15.0%)	54 (24.8%)	15 (14.3%)
5–6	24 (6.9%)	24 (11.1%)	2 (1.9%)
Biochemistry on admission			
Hemoglobin, n (%) below reference			
Female ≤ 7.3 mmol/L	77 (20.5%)	47 (21.8%)	77 (6.7%)
Male ≤ 8.3 mmol/L			
Hematocrit, n (%) below reference			
Female ≤ 0.35 fraction	76 (20.5%)	49 (22.9%)	8 (7.7%)
Male ≤ 0.4 fraction			
Platelets, n (%) below reference			
Female ≤ 165 × 10^9^/L	25 (6.6%)	16 (7.4%)	4 (3.8%)
Male ≤ 145 × 10^9^/L			
International Normalized Ratio, n (%) above reference			
Female/Male ≤ 1.2	57 (15.1%)	38 (17.4%)	11 (10.5%)
Activated partial thromboplastin time, n (%) above reference			
Female/Male ≥ 29 s	23 (6.2%)	17 (8.1%)	3 (2.9%)

### Agreement

The agreement between GFAP/UCH-L1 and S100B was high in the three cohorts (Table 2). Cohens kappa improved with increasing proximity to clinical biomarker use and reached an almost perfect identity in sub-cohort 2. Patients with non-agreeing biomarker levels in each of the three scenarios are presented in Table 3. Generally, these patients had biomarkers levels close to the decision cutoffs. In the unselected trauma cohort, few of the S100B positive/GFAP/UCH-L1 negative patients had a head trauma described in the admission chart and these patients also had a low ISS score. Two patients with positive head-CT were not detected by the S100B assay in the unselected trauma cohort and sub-cohort 1. These were older (> 65 years) males how both had an ISS score of 25 and GCS of 3 and 4.

**Table 2:** Agreement and diagnostic performance of GFAP/UCH-L1 and S100B

	Agreement	Cohens kappa	Sensitivity		Specificity		Positive predictive value		Negative predictive value	
			GFAP/UCH-L1 (%)	S100B (%)	GFAP/UCH-L1 (%)	S100B (%)	GFAP/UCH-L1 (%)	S100B (%)	GFAP/UCH-L1 (%)	S100B (%)
Unselected trauma cohort	89.7	0.69 (0.60 to 0.78) *p* < 0.0001	100	97.5	24.4	26.3	28.7	28.3	100	97.2
Sub-cohort 1	93.6	0.70 (0.56 to 0.84) *p* < 0.0001	100	97.4	15.9	21.0	40.2	41.1	100	93.6
Sub-cohort 2	92.4	0.77 (0.62 to 0.92) *p* < 0.0001	100	100	22.9	27.6	20.2	21.3	100	100

Each parameter was analyzed in three cohorts of increasing proximity to clinical biomarker use. The unselected trauma cohort included all patients (n = 379). Sub-cohort 1 included patients with evident head trauma as described in the admission chart (n = 218). Sub-cohort 2 included patients with evident head trauma, ISS ≤ 15, GCS ≥ 14, and blood sampling within 6 h (S100B) or 12 h (GFAP/UCH-L1) from the injury (n = 105)

**Tabel 3:** Characteristics of patients with dis-agreeing samples in the three models analyzed

	Unselected trauma cohort		Sub-cohort 1		Sub-cohort 2	
	GFAP/UCH-L1 positive S100B negative	S100B positive GFAP/UCH-L1 negative	GFAP/UCH-L1 positive S100B negative	S100B positive GFAP/UCH-L1 negative	GFAP/UCH-L1 positive S100B negative	S100B positive GFAP/UCH-L1 negative
N	23	16	12	2	6	2
Age years, mean ± SD	47.6 ± 18.6	35.2 ± 12.8	49.6 ± 22.7	33.0 ± 15.6	43.5 ± 23.9	33.0 ± 15.6
Sex (fm/m)	9/14	7/9	5/7	0/2	5/1	0/2
Time from injury to admission, mins median (IQR)	77.0 (42 to 115)	42.5 (31 to 68)	77 (42 to 83)	44.5 (38 to 51)	54.5 (39 to 79)	44.5 (38 to 51)
Admission delay > 6 h	3	0	1	0	0	0
Admission delay > 12 h	2	0	1	0	0	0
GCS, mean ± SD (min–max)	13.7 ± 3.3 (3–15)	14.9 ± 0.25 (14–15)	12.6 ± 4.4 (3–15)	14.5 ± 0.7 (14–15)	14.8 ± 0.4 (14–15)	14.5 ± 0.7 (14–15)
ISS, mean ± SD (min–max)	10.7 ± 10.5 (1–29)	3.9 ± 4.3 (1–13)	9.9 ± 10.2 (1–25)	1*	3.7 ± 4.4 (1–12)	1 ± 0 (1 to 1)
GFAP, ng/L, median IQR)	47.7 (25.2 to 133.2)	15.5 (13.1 to 219)	61.1 (43.6 to 120.7)	19.3 (17.1 to 21.4)	47.2 (40.5 to 91.6	19.3 (17.1 to 21.4)
UCHL1, ng/L, median (IQR)	390.6 (180.3 to 658.1)	292.8 (254.4 to 362.1)	224.3 (176.6 to 362.2)	339.1 (282.2 to 395.9)	217.5 (180.3 to 390.6)	339.1 (282.2 to 395.9)
S100B, µg/L, median (IQR)	0.056 (0.043 to 0.075)	0.214 (0.131 to 0.406)	0.057 (0.045 to 0.065)	0.159 (0.104 to 0.215)	0.059 (0.054 to 0.068)	0.159 (0.104 to 0.215)
Head trauma in chart	12	2	12	2	6	2
Head-CT lesion	2	0	2	0	0	0

The unselected trauma cohort included all patients (n = 379). Sub-cohort 1 included patients with evident head trauma as described in the admission chart (n = 218). Sub-cohort 2 included patients with evident head trauma, ISS ≤ 15, GCS ≥ 14, and blood sampling within 6 h (S100B) or 12 h (GFAP/UCH-L1) from the injury (n = 105). Data presented as absolute numbers unless otherwise indicated. Abbreviations: SD: standard deviation; IQR: interquartile range, ISS: Injury severity score; min: minimum value, max: maximum value; GCS: Glasgow coma scale; GFAP: Glial Fibrillary Acidic Protein; UCHL1: Ubiquitin carboxy-terminal hydrolase L1; S100B: S100 Calcium-binding protein B. *1 in both samples

### Diagnostic performance

The diagnostic performance characteristics of GFAP/UCH-L1 and S100B are presented in Table 2. The GFAP/UCH-L1 had a slightly higher sensitivity and negative predictive value than S100B in the unselected trauma cohort and sub-cohort 1. Conversely, the specificity was slightly higher for S100B than for GFAP/UCH-L1 in these models. Yet, in sub-cohort 2 the analyses had comparable performances.

### Assay evaluation

The distribution of positivity/negativity of the TBI® assay components (GFAP/UCH-L1) were evaluated in the unselected trauma cohort as well as in patients with positive head-CT. In both scenarios, positive TBI-assay test results occurred from single positivity of GFAP or UCH-L1 (Supplemental Tables 1 and 2). Quantitative levels of each TBI-assay component were compared with levels measured by Simoa (Supplemental Fig. 1 and 2). This revealed a low concordance (GFAP = 0.345 and UCHL1 = 0.153) and a high bias [GFAP = 0.35 (95% CI 0.34–0. 37) and UCH-L1 = 1.834 (95% CI 1.82–1.86).

## Discussion

This study demonstrates an almost perfect agreement between S100B and GFAP/UCH-L1 when applied on a Scandinavian trauma population. Additionally, it confirms S100B and GFAP/UCH-L1 to have comparable diagnostic performances for detecting traumatic intracranial lesions in adult mTBI patients. This supports GFAP/UCH-L1 as a feasible alternative to S100B in clinical decision-making in mTBI, but with less contraindications, which may hold potential to increase mTBI guideline-adherence.

We performed a head-to-head comparison of the serum-based S100B single test and the plasma-based GFAP/UCH-L1 combination test in a Scandinavian trauma cohort by estimating classification agreement by Cohens Kappa. Our results revealed an almost perfect agreement that increased in sub-cohorts with proximity to clinical biomarker use. The few misclassified patients had biomarker levels close to the decision cut-offs, and the two patients with positive head-CT that were missed by S100B in the unselected trauma cohort and the sub-cohort 1 were excluded by the applied criteria for the sub-cohort 2. Our results therefore demonstrate a high level of agreement between S100B and GFAP/UCH-L1 when used according to their indication. Despite considerable research evaluating S100B and GFAP/UCH-L1, a direct comparison between prior studies is hampered by differences in cohorts, sample times, cutoffs, serum vs plasma, and assay used [14, 16, 31, 32]. To allow a more direct comparison with prior studies, we evaluated the diagnostic performance of S100B and GFAP/UCH-L1 separately in all cohorts. In sub-cohort 2, S100B had a sensitivity and a negative predictive value as reported in prior studies [7, 8, 31, 33, 34]; however, when applied more broadly among the unselected trauma cohort and the sub-cohort 1, the performance declined. By contrast, GFAP/UCH-L1 had a sensitivity and a negative predictive value of 100% across all three cohorts, which is also comparable to prior studies [14, 17, 31]. This difference in diagnostic performance seems to result from assay sensitivity to extracranial injuries, thereby highlighting the broader applicability of GFAP/UCH-L1 compared with S100B. In terms of specificity, we observed relatively low levels for both S100B and GFAP/UCH-L1. Prior studies report specificities for S100B ranging from 47 to 53% [8, 35] and for GFAP/UCH-L1 from 28.8 to 39% [17, 18, 36]. Thus, these tests are associated with a high number of false positives, which may explain some of the challenges with clinical implementation of S100B [8]. It is, however, well established that S100B and GFAP levels increase with age [37], and establishment of age-specific cutoffs would likely improve the performance of these biomarkers substantially.

The SNC guidelines for management of head trauma recommend use of S100B to determine the need for head-CT in patients with low-risk mTBI i.e. GCS 14 or GCS 15 and suspected/confirmed loss of consciousness and/or ≥ 2 vomiting episodes within 6 h from injury [7]. Yet, non-adherence to the SNC guidelines results in an unacceptably low sensitivity [6, 8, 38], and is associated with unnecessary head-CT, increased health care costs and prolonged emergency department time [8, 39, 40]. The causes of non-adherence is not clear, but might be associated with clinical judgment, anamnestic uncertainty, unclear symptoms, prolonged analysis time of serum tests, and the extracranial sources of S100B and the short half-life of S100B, which lower the number of patients suitable for testing [20, 39]. Despite being recommended in the SNC guidelines, S100B has not achieved FDA approval. The FDA has, however, approved GFAP/UCH-L1 for clinical use, and the Spanish and French guidelines recommend GFAP/UCH-L1 for clinical use as it may offer a feasible alternative to S100B [17, 19–21]. It may be used for up to 12 h after injury, it is not susceptible to extracranial injuries, and it is a plasma-based analysis, which can be performed more rapidly by the clinical laboratory. Whether these improvements will lead to increased use remains unknown and require further studies. Yet, our data clearly suggest that GFAP/UCH-L1 can safely be incorporated as part of clinical care which will facilitate progression of studies to evaluate if this will translate into clinical and economic benefits.

The necessity of a combination test with GFAP and UCH-L1 compared to a single test with S100B remains controversial [14]. Some studies suggest that GFAP and UCH-L1 complement each other for the detection of traumatic intracranial lesions [41, 42], while others favor GFAP or UCH-L1 alone for good diagnostic performance [13–15, 36, 41, 43, 44]. In our study, positivity depended on GFAP or UCH-L1, and not on GFAP or UCH-L1 alone. These controversies might stem from biomarker kinetics, and therefore critically dependent on research setting and intended use of the biomarkers. While UCH-L1 peaks immediately after injury and decline rapidly, GFAP peaks later after injury and remains elevated for a prolonged period of time [16, 45]. Thus, sampling later after injury would tend to favor the GFAP component and vise-versa [46, 47]. The decision to exclude one of the assay components should therefore be made with careful respect to the clinical setting, and for most Scandinavian centers with mixed populations, the combination assay would offer the safer alternative.

A key aspect in comparing TBI biomarker studies is the assay used. GFAP and UCH-L1 are complex proteins that are expressed in several isoforms, and they may therefore be differentially detected by the assay available [48, 49]. Use of different assays can therefore be of great importance for the obtained results and their interpretation. To provide comparability of data to the literature, we compared GFAP and UCH-L1 measured on the Alinity-I platform and the Simoa platform [50]. This revealed a low concordance with the Alinity-I platform measuring GFAP levels on average 65% lower and UCH-L1 levels 84% higher than the Simoa platform. Such differences between assays are commonly and frequently encountered, especially at early stages of development. It demonstrates the need for caution in comparison of studies using different methods as well as the need for assay-specific cutoffs.

There are limitations to the study that needs to be taken into consideration. Most importantly, the cohort investigated was collected for biomarker studies, but not specifically for this study [23, 24]. The population therefore consisted of patients admitted to the trauma center, but excluded patients admitted to the emergency department who would also be candidates for GFAP/UCH-L1 evaluation. Yet, the primary endpoint was classification agreement, which is best done including the entire spectrum of disease, and the secondary endpoint showed increased biomarker performance when the cohort characteristics approached that of clinical biomarker use. The classification of patients in the two sub-cohorts was based on a retrospective chart review and we cannot exclude the possibility of some classification bias and inaccuracies regarding the secondary endpoint. It would, however, be equal for S100B and GFAP/UCH-L1, and therefore have limited impact on the study. Due to these potential risks of classification problems, we also refrained from grouping of the mTBI patients into SNC risk-groups. Finally, the interpretation of Cohens Kappa has been debated as to which levels should be considered high. However, the levels reached in our study was very close to the maximum obtainable value and are indisputably very high.

## Conclusions

The S100B single-test and the GFAP/UCH-L1 combination-test have almost perfect agreement for classification of patients with mTBI. Both assays have sensitivities and negative predictive values for detecting traumatic intracranial lesions of 100% in our cohort of adult Scandinavian trauma patients. These data support GFAP/UCH-L1 as a feasible alternative to S100B for evaluation of patients with mTBI.

## Supplementary Information

Additional file 1.

## Data Availability

No datasets were generated or analysed during the current study.

## Abbreviations

CT: Computed tomography
GCS: Glasgow Coma scale
GFAP: Glial fibrillary acidic protein
ISS: Injury severity score
IQR: Interquartile ranges
mTBI: Mild traumatic brain injury
SNC: Scandinavian Neurotrauma Committee
Simoa: Single molecule array
SD: Standard deviation
S100B: S100 astroglial calcium-binding protein B
TBI: Traumatic brain injury
UCH-L1: Ubiquitin C-terminal hydrolase
FDA: US Food and Drug Administration
CI: 95% Confidence intervals
