# Supplementary material for: A head-to-head comparison of S100B and GFAP/UCH-L1 for detection of traumatic intracranial lesions in a Scandinavian trauma cohort
Source: Scand J Trauma Resusc Emerg Med. 2025 Mar 25;33:52. doi: 10.1186/s13049-025-01364-9 (PMC11938562; doi:10.1186/s13049-025-01364-9)
Supplement: Supplementary file 1 — Additional file 1. [file 13049_2025_1364_MOESM1_ESM.docx]

Title

A head-to-head comparison of S100B and GFAP/UCH-L1 for detection of traumatic intracranial lesions in a Scandinavian trauma cohort.

*Full names and institutional addresses for all authors*

Thea Overgaard Wichmann, MD

^1^Department of Neurosurgery – CENSE-Spine

Aarhus University Hospital, Aarhus, Denmark

^2^Department of Surgery and Intensive Care

Viborg Regional Hospital, Viborg Denmark

Thewic@rm.dk

Ayad Babaee, B.Sc

^3^Department of Clinical Biochemistry

Aalborg University Hospital, Aalborg, Denmark

Ayadbabaee@gmail.com

Kirsten Duch, Cand.Scient

^4^Research Data and Biostatistics

Aalborg University Hospital, Aalborg, Denmark

K.duch@rn.dk

Mikkel Mylius Rasmussen, MD PhD

^1^Department of Neurosurgery – CENSE-Spine

Aarhus University Hospital, Aarhus, Denmark

^5^Department of Clinical Medicine

Aarhus University, Aarhus, Denmark

Mikkrasm@rm.dk

Maj Lesbo, MD

^6^Department of Orthopaedic Surgery

Viborg Regional Hospital, Viborg, Denmark

Maj.lesbo@rm.dk

Ole Brink, MD

^7^Department of Orthopaedic Surgery

Aarhus University Hospital, Aarhus, Denmark

Olebrink@rm.dk

Lars C. Borris, MD

^7^Department of Orthopaedic Surgery

Aarhus University Hospital, Aarhus, Denmark

Larsborris65@gmail.comLarsborris65@gmail.com

Claus V. B. Hviid, MD PhD

^3^Department of Clinical Biochemistry

Aalborg University Hospital, Aalborg, Denmark

^8^Department of Clinical Medicine

Aalborg University, Aalborg, Denmark

Claus.hviid@rn.dk

*Corresponding author*

Thea Overgaard Wichmann, MD

^1^Department of Neurosurgery – CENSE-Spine

Aarhus University Hospital, Aarhus, Denmark

^2^Department of Surgery and Intensive Care

Viborg Regional Hospital, Viborg Denmark

Thewic@rm.dk

Content

Table 1: Agreement of GFAP/UCH-L1 components in all patients

Table 2: Agreement of GFAP/UCH-L1 components in patients with positive head-CT

Figure 1: Comparison of Alinity and Simoa, GFAP-component

Figure 2: Comparison of Alinity and Simoa, UCH-L1-component

**Table 1. Agreement of GFAP/UCH-L1 assay components in all patients**

|  |  | UCH-L1 | | |
| --- | --- | --- | --- | --- |
|  |  | Negative | Positive | missing |
| GFAP | Negative | 78 | 66 | 0 |
|  | Positive | 18 | 194 | 1 |
|  | Missing | 0 | 22 | 0 |

Results of each component of the GFAP/UCH-L1 assay in all patients (n=379). The test result is returned as positive, negative, or inconclusive based on the combined results of the assay components. Positivity of either GFAP and/or UCH-L1 will result in a positive test result. The test is negative when both components are negative. Combinations of negative/missing or missing/missing are inconclusive. The assay specific cut-offs are <400 ng/L for UCH-L1 and <35 ng/L for GFAP.

**Table 2. Agreement of GFAP/UCH-L1 assay components in patients with positive head-CT**

|  |  | UCH-L1 | | |
| --- | --- | --- | --- | --- |
|  |  | Negative | Positive | Missing |
| GFAP | Negative | 0 | 1 | 0 |
|  | Positive | 3 | 72 | 0 |
|  | Missing | 0 | 5 | 0 |

Results of each component of the GFAP/UCH-L1 assay in patients with positive head-CT (n=81). The test result is returned as positive, negative, or inconclusive based on the combined results of the assay components. Positivity of either GFAP and/or UCH-L1 will result in a positive test result. The test is negative when both components are negative. Combinations of negative/missing or missing/missing are inconclusive. The assay specific cut-offs are <400 ng/L for UCH-L1 and <35 ng/L for GFAP.

**Figure 1. Comparison of the GFAP/UCH-L1 assay with Simoa, GFAP-component**


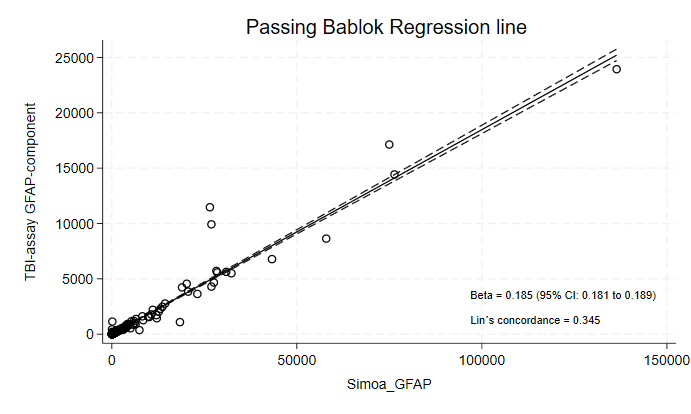


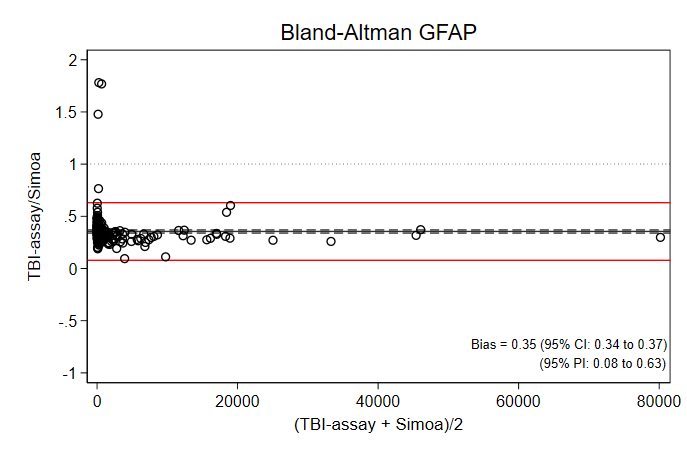


Comparison of the GFAP levels measured by the Alinity and the Simoa technology. Measured values were compared by Passing Bablok regression (upper graph) and in a Bland-Altman plot (lower graph). The regression line with 95% confidence intervals and the individual values are displayed along with the beta-coefficient and Lin´s concordance correlation coefficient. The Bland-Altman plot shows relative (Alinity/Simoa) values against average biomarker level. The bias with 95% confidence interval is displayed with black punctured line and the 95% prediction interval is indicated by red lines. The line of identify (Alinity/Simoa = 1) is shown by dotted line. The bias with 95% confidence and prediction interval is also displayed in lower right corner.

**Figure 2: Comparison of the GFAP/UCH-L1 assay with Simoa, GFAP-component**


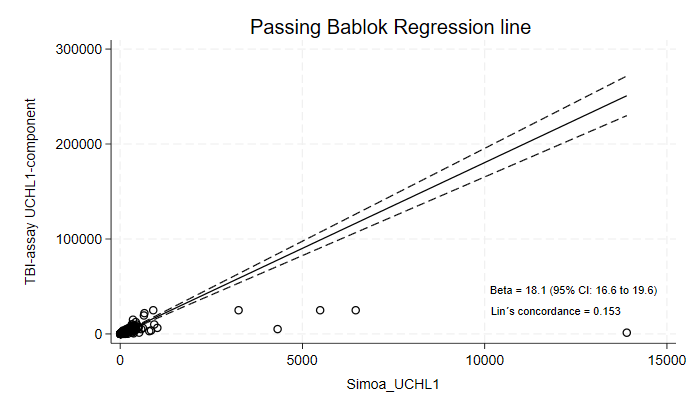


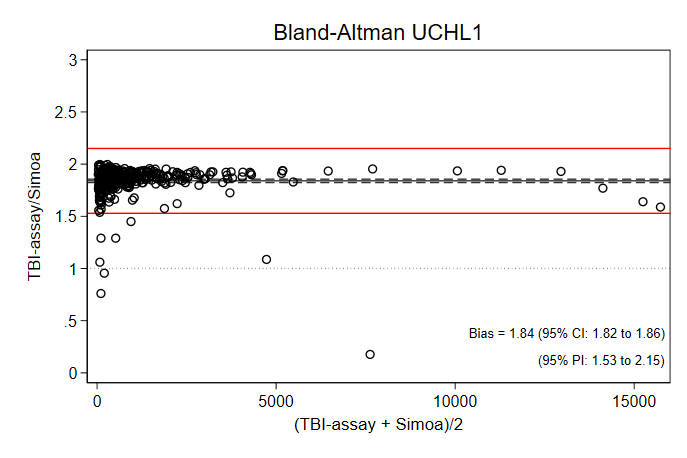


Comparison of the UCHL1 levels measured by the Alinity and the Simoa technology. Measured values were compared by Passing Bablok regression (upper graph) and in a Bland-Altman plot (lower graph). The regression line with 95% confidence intervals and the individual values are displayed along with the beta-coefficient and Lin´s concordance correlation coefficient. The Bland-Altman plot shows relative (Alinity/Simoa) values against average biomarker level. The bias with 95% confidence interval is displayed with black punctured line and the 95% prediction interval is indicated by red lines. The line of identify (Alinity/Simoa = 1) is shown by dotted line. The bias with 95% confidence and prediction interval is also displayed in lower right corner.
